# Point-of-care Ultrasound Detection of Cataract in a Patient with Vision Loss: A Case Report

**DOI:** 10.5811/cpcem.2020.4.46597

**Published:** 2020-07-14

**Authors:** Kyle Dornhofer, Marawan Alkhattabi, Shadi Lahham

**Affiliations:** University of California, Irvine Medical Center, Department of Emergency Medicine, Orange, California

**Keywords:** POCUS, ultrasonography, ocular, cataract, vision loss

## Abstract

**Background:**

Point-of-care ocular ultrasound in the emergency department (ED) is an effective tool for promptly evaluating for several vision-threatening etiologies and can be used to identify more slowly progressing etiologies as well, such as cataract formation within the lens.

**Case Report:**

A 62-year-old female presented to the ED with a two-day history of painless vision loss of the left eye as well as reduced vision for the prior 30 days.

**Conclusion:**

Point-of-care ultrasound was performed and showed calcification of the lens consistent with cataract.

## INTRODUCTION

Vision loss is a common complaint encountered in the emergency department (ED) and frequently prompts further imaging or consultation. Point-of-care ultrasound (POCUS) is well suited for the rapid assessment of several potential etiologies ranging from acutely vision-threatening to slowly progressive or chronic. We present a case of an elderly female with vision loss who was evaluated with POCUS and found to have a cataract, a diagnosis rarely initially made in the ED. In this case, point-of-care ocular ultrasound rapidly guided further management and disposition.

## CASE REPORT

A 62-year-old female presented to the ED with a two-day history of painless vision loss of the left eye. She reported gradual reduction in her vision over the prior 30 days, with a more dramatic reduction in her vision over the prior two days. She denied any eye pain, flashers, floaters, or diplopia. Past medical history was significant for cerebrovascular accident, hypertension, type 2 diabetes mellitus, and hypercholesterolemia. In the ED, physical exam was significant for chronic left facial droop with white opacification of the left lens, a visual acuity of 20/30 in the right eye, and perception of light only in the left eye. Point-of-care ocular ultrasound using a linear probe (10 megahertz) in the ocular setting showed lens calcification consistent with cataract (Image).

Ophthalmology was consulted, and an anterior segment exam was performed. The right eye showed a diffuse grade 4 superficial punctate keratitis (SPK) with a grade 2 nuclear sclerotic cataract. In contrast, the left eye showed a grade 3 SPK with grade 4 mature cataract. The funduscopic exam was normal in the right eye, while the left eye view was obscured due to the mature cataract. B-scan was performed by ophthalmology, again showing a significant cataract in the left eye. Outpatient follow-up with possible cataract extraction with intraocular lens implantation was recommended by ophthalmology.

## DISCUSSION

Cataract is a clouding of the crystalline lens inside the eye. It is the leading cause of blindness and the most prevalent ocular disease worldwide.^1^,^2^ In the United States, it is the third leading cause of treatable blindness.^3^,^4^ Cataracts typically occur gradually as a result of aging or secondary to trauma, inflammation, metabolic/nutritional disorders, or radiation, with age-related cataracts being the most common cause.^5^,^6^ Cataracts are considered one of the earliest complications of diabetes mellitus.^7^

Patients often present with gradually decreased vision and increased problems with glare. They may or may not experience changes in refractive error and loss of stereopsis (depth perception). In the ED the ocular exam should include pupil examination, assessment of extraocular muscles, measurement of visual acuity and intraocular pressure, and confrontational visual field testing.^8^,^9^ Measurement of visual acuity under both high and low illumination can be helpful. Definitive diagnosis is made with slit lamp examination and direct visualization of the cataract within the lens. Treatment consists of vision correction with lenses or surgery depending on the severity of the cataract.^7^

## CONCLUSION

Point-of-care ocular ultrasound can be performed when there is concern for posterior globe pathology (i.e., retinal/vitreous detachment), but visualization of the back of the eye with the slit lamp is obscured due to an opaque lens or when eyelids are swollen shut following injury. Indications for ocular ultrasound include decreased vision/loss of vision, suspected foreign body, ocular pain, eye trauma, and head injury. POCUS provides a rapid and noninvasive evaluation for several vision-threatening ocular emergencies including globe perforation, retrobulbar hematoma, retinal detachment, lens subluxation, vitreous hemorrhage, and intraocular foreign body.^10^–^12^ Occasionally, as in this case, cataract formation within the lens can be directly visualized with ultrasound.

**CPC-EM Capsule**

What do we already know about this clinical entity?

Cataract is the leading cause of blindness and the most prevalent ocular disease worldwide. Vision loss is a common complaint encountered in the emergency department.

What makes this presentation of disease reportable?

Point-of-care ultrasound (POCUS) was performed in a patient complaining of vision loss and showed findings consistent with cataract.

What is the major learning point?

POCUS is an effective tool for promptly evaluating a patient with complaints of vision loss.

How might this improve emergency medicine practice?

Ultrasound provides a rapid and noninvasive evaluation for several vision-threatening ocular emergencies.

## Figures and Tables

**Image:**
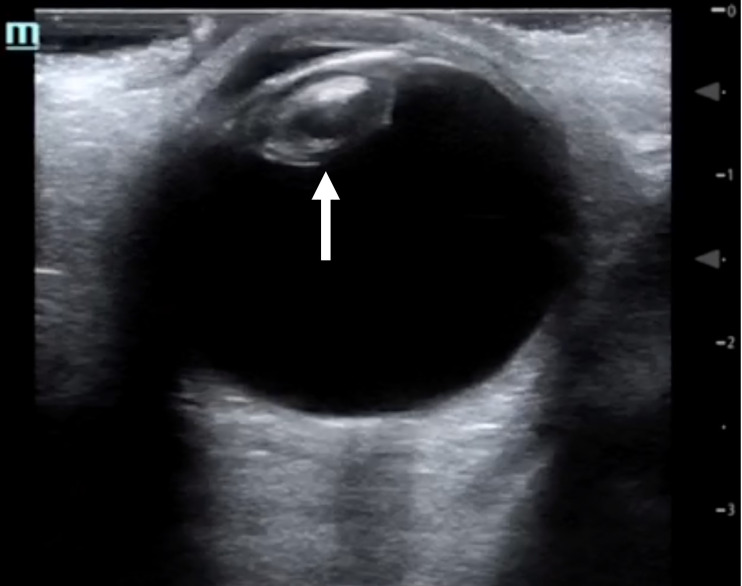
Point-of-care ocular ultrasound image in the transverse plane, revealing lens calcification consistent with cataract (arrow).
